# Evaluation of serum prolidase enzyme activity and oxidative stress in patients with tinnitus^☆^

**DOI:** 10.1016/j.bjorl.2019.01.009

**Published:** 2019-03-15

**Authors:** Adnan Ekinci, Kaan Kamasak

**Affiliations:** a Hitit University, Faculty of Medicine, Otorhinolaryngology Department, Çorum, Turkey; b Hitit University, Faculty of Medicine, Neurosurgery Department, Çorum, Turkey

**Keywords:** Tinnitus, Prolidase, Oxidative stress, Zumbido, Prolidase, Estresse oxidativo

## Abstract

**Introduction:**

Tinnitus is defined as the perception of sound in the head or in the head in the absence of external sounds. The cause of tinnitus is still unknown.

**Objective:**

We aimed to compare the serum levels of total oxidant status, total antioxidant status, serum prolidase enzyme activity and the oxidative stress index in patients with tinnitus to those of normal subjects.

**Methods:**

Twenty five patients with tinnitus (mean age 34.3) and 25 healthy controls (mean age 37.2) were included in the study.

**Results:**

Total oxidant status levels in the patient group were significantly higher than in the control group (*p* = 0.037). The mean total oxidant status value was 2.54 ± 0.95 mmoL/L in the patient group, and 2.06 ± 0.98 mmoL/L in the control group. The mean oxidative stress index level was 0.22 ± 0.10 AU in the patient group, while it was 0.17 ± 0.08 AU in the control group. Oxidative stress index was significantly higher in the patient group (0.026). There was no significant difference between the groups in terms of total antioxidant status values (*p* = 0.838). The mean serum prolidase enzyme activity level was 202.74 ± 33.56 U/L in the patient group and 175.46 ± 42.68 U/L in the control group. Serum prolidase enzyme activity levels in the patient group were significantly higher than in the control group (0.040).

**Conclusion:**

We detected that the total oxidant status, oxidative stress index and serum prolidase enzyme activity levels were higher in patients with tinnitus when compared to the healthy controls. This finding suggests that oxidative stress index and serum prolidase enzyme activity may play a role in the etiopathogenesis of tinnitus.

## Resumo

**Introdução:**

O zumbido é definido como a percepção de um som na cabeça na ausência de sons externos. A causa do zumbido ainda é desconhecida.

**Objetivo:**

Comparar os níveis séricos do estado oxidante total, estado antioxidante total, atividade da enzima prolidase sérica e o índice de estresse oxidativo em pacientes com zumbido e em indivíduos normais.

**Método:**

Foram incluídos no estudo 25 pacientes com zumbido (média de 34,3 anos) e 25 controles saudáveis (média de 37,2 anos).

**Resultados:**

Os níveis do estado oxidante total no grupo de pacientes foram significantemente maiores do que no grupo controle (*p* = 0,037). O valor médio do estado oxidante total foi de 2,54 ± 0,95 mmoL/L no grupo de pacientes e de 2,06 ± 0,98 mmoL/L no grupo controle. O nível médio de índice de estresse oxidativo foi de 0,22 ± 0,10 AU no grupo de pacientes, enquanto no grupo controle foi de 0,17 ± 0,08 AU. O índice de estresse oxidativo foi significantemente maior no grupo de pacientes (0,026). Não houve diferença significante entre os grupos em relação aos valores do estado antioxidante total (*p* = 0,838). O nível médio de atividade da enzima prolidase sérica foi de 202,74 ± 33,56 U/L no grupo de pacientes e de 175,46 ± 42,68 U/L no grupo controle. Os níveis de atividade da enzima prolidase sérica no grupo de pacientes foram significantemente maiores do que no grupo controle (0,040).

**Conclusão:**

Detectamos que os níveis de estado oxidante total, índice de estresse oxidativo e atividade da enzima prolidase sérica foram maiores nos pacientes com zumbido quando comparados aos controles saudáveis. Esse achado sugere que o índice de estresse oxidativo e a atividade da enzima prolidase sérica podem desempenhar um papel na etiopatogenia do zumbido.

## Introduction

Tinnitus is defined as the perception of sound in the head or in the head in the absence of external sounds. Tinnitus is among the most common symptoms of the hearing system and adversely affects the quality of life of patients.^1^ The incidence of tinnitus is between 10% and 15%.^2^ Tinnitus has been reported to be caused by a number of diseases affecting the auditory nervous system. However, the cause of tinnitus is still unknown.^3^

Prolidase is a metalloenzymide belonging to the family of hydrolases, which plays an important role in collagen degradation and biosynthesis. Prolidase enzymatic activity has been detected in many tissues such as stomach, heart, brain, pancreas, kidney, liver and amniotic fluid.^4^ It is thought that changes in the activity of prolidase enzyme may play a role in the development of many diseases such as nasal polyps, schizophrenia, anxiety, bipolar disorder, major depression, Type2 diabetes mellitus, chronic hepatitisC, osteoporosis, bronchial asthma and liver fibrosis.^5^, ^6^, ^7^, ^8^, ^9^, ^10^, ^11^

Reactive oxygen species (ROS) are reactive molecules containing one or more unpaired electrons in their outer atomic orbitals.^12^ ROS overproduction is involved in the pathogenesis of many diseases. These include diabetes mellitus, cancer, rheumatoid arthritis, systemic lupus erythematosus, Behçet's disease, and atherosclerosis.^13^ Various antioxidant defense mechanisms have been developed by the organism to protect against the adverse effects of ROS. Under normal conditions there is a balance between the antioxidant defense mechanism and free radicals, but oxidative stress is observed if this balance is impaired in the direction of free radicals.^14^ Oxidant and antioxidant molecules can be measured individually in the plasma but recently total oxidant status (TOS) and total antioxidant status (TAS) measurements have been improved.^15^

We decided to do this study because of the fact that oxidative stress and prolidase enzyme are being investigated in many diseases and there is a limited number of studies addressing their relationships with etiopathogenesis of tinnitus, which in turn, is still not fully explained. We aimed to compare the serum levels of prolidase, TAS and TOS in tinnitus patients with normal healthy subjects. Although the relationship between tinnitus and oxidative stress has been studied previously, its relationship with prolidase has never been investigated. This is the first study in the literature to examine the levels of prolidase enzymes in patients with tinnitus.

## Materials and methods

This study was carried out at the Otorhinolaryngology Clinic of Hitit University between 10 January 2018 and 31 March 2018. The study protocol was approved by Hitit University, Ethics Committee for Clinical Research (decree no. 2018-23). A total of 25 patients diagnosed with tinnitus and 25 healthy subjects were included in the study. There were 14 were males and 11 were females with a mean age of 34.3 years in the tinnitus group. The control group consisted of 25 subjects, 12 males and 13 females, with a mean age of 37.2 years. All participants provided their written informed consents. All patients were examined by an otolaryngologist with detailed history. In addition, routine blood tests, audiologic evaluation and neuro-otologic diagnostic evaluation were performed. The criteria for inclusion in the tinnitus group were the presence of subjective tinnitus for at least 1 year at the age of 18–65 years and no medical treatment for tinnitus until at least 8 weeks before entering the study. The control group included normal healthy individuals who had no complain of tinnitus, between the ages of 18–65, assisted in our clinic for other reasons. In both groups, the exclusion criteria were the presence of otosclerosis, tympanosclerosis, chronic otitis media, vestibular schwannomas, Meniere's disease, malignancy, autologous surgical history, venous hum, ototoxic drug taking, temporal bone trauma, middle or external ear problems. Patients with metabolic, hematologic, cardiovascular, psychiatric and neurological diseases were also excluded from the study. Otologic, neurological and audiologic examinations were normal for all participants. Only one serum sample was taken before any type of treatment, from patients and healthy volunteers. An informed consent was obtained from all patients in both groups as an indication of their voluntary participation in the study. A 10 mL of venous blood sample was drawn from each patient between 8:00 AM and 10:00 AM, into the clot activator containing tubes (Isotherm, Hongyu Medical, Weihai, China) after 12 h of fasting. After the formation of the blood clot, the samples were centrifuged for 10 min at 4000 rpm, and the serum was separated and then stored at −80 °C until the day of analysis.

### TAS, TOS and prolidase measurements

We used total antioxidant and total oxidant capacity measurements, developed by Erel.^15^, ^16^ For the TAS and TOS analyzes, the method developed by Erel was used. For this purpose a completely automated RelAssay kit (Rel Assay Diagnostics kit, Mega Tip, Gaziantep, Turkey) was used and colorimetric method using the VitalScientific, Selectra/Flexor E (The Netherlands) autoanalyzer. TAS and TOS Results were presented as μmoL H_2_O_2_ equivalent/L. SPEA levels were measured by ELISA (Cat. no. CSB-E16196 h, Cusabo Biotech Co. Ltd). Intra-assay Coefficient of Variation (CV) and inter-assay coefficient were <8% and <10%, respectively. The test interval was between 93.75 and 6000 mU/mL with a sensitivity of 93.75 mU/mL. For the measurements, Radim company ALİSEİ automatic ELISA was used.

### Statistical analysis

The SPSS software package (version 22.0, SPSS Inc., Chicago, IL, USA) was chosen for statistical analysis. The normality distribution was determined by the Shapiro–Wilk test. Continuous variables were presented as mean ± standard deviation, median (min–max) according to dispersion hypotheses, and categorical variables presented as number and percentage. When continuous variables were assessed, two independent factors were analyzed by Student's *t*-test if the variables were normally distributed. The Mann–Whitney *U* test was used when independent samples were not normally distributed. Significance level was accepted as p < 0.05.

## Results

**Figure 1:**
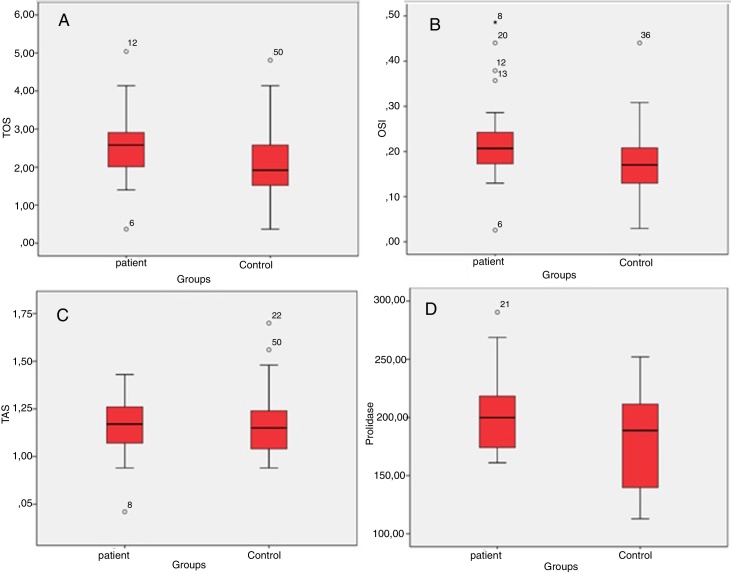
Box plots of mean changes in TOS (A), OSİ (B), TAS (C) and SPEA (D) according to groups.

The patient and the control groups were similar in terms of age and gender (*p *> 0.05 for both). The mean TOS value was 2.54–0.95 mmoL/L and median (min–max) 2.58 (0.37–5.04 mmoL/L) in the patient group, and it was 2.06 ± 0.98 mmoL/L and median (min–max) 1.92 (0.37–4.81 mmoL/L) in the control group. Serum TOS levels in the patient group were significantly higher than in the control group (*p* = 0.037) (Table 1, Fig. 1A). The mean OSI levels was 0.22 ± 0.10 AU and median (min–max) 0.20 (0.03–0.49 AU) in the patient group, while it was 0.17 ± 0.08 AU and median (min–max) 0.17 (0.03–0.44 AU) in the control group. OSI was significantly higher in the patient group (0.026) (Table 1, Fig. 1B). The mean TAS level was 1.16 ± 0.15 mmoL/L and median (min–max) 1.17 (0.71–1.43 mmoL/L) in the patient group and 1.18 ± 0.18 mmoL and median (min–max) 1.15 (0.94–1.70 mmoL/L) in the control group. There was no significant difference between the groups in terms of TAS values (*p* = 0.838) (Table 1, Fig. 1C). The mean SPEA level was 202.74 ± 33.56 U/L and median (min–max) 199 (161–290 U/L) in the patient group and 175.46 ± 42.68 U/L and median (min–max) 188 (112–251 U/L) in the control group. SPEA levels in the patient group were significantly higher than the control croup (0.040) (Table 1, Fig. 1D).

**Table 1:** Comparison of patients and control groups in terms of TOS, OSİ, TAS and SPEA levels.

Group		*n*	Mean ± SD	Median (min–max)	*p*-value
TOS	Patient	25	2.54 ± 0.95	2.58 (0.37–5.04)	0.037^a^
	Control	25	2.06 ± 0.98	1.92 (0.37–4.81)	
OSİ	Patient	25	0.22 ± 0.10	0.20 (0.03–0.49)	0.026^a^
	Control	25	0.17 ± 0.08	0.17 (0.03–0.44)	
TAS	Patient	25	1.16 ± 0.15	1.17 (0.71–1.43)	0.838
	Control	25	1.18 ± 0.18	1.15 (0.94–1.70)	
SPEA	Patient	25	202.78 ± 33.56	199 (161–290)	0.040^a^
	Control	25	175.46 ± 42.68	188 (112–251)	

SD, standard deviation; min, minimum; max, maximum.

a Statistically significant (*p* < 0.05).

## Discussion

Tinnitus is classified as objective and subjective. Objective tinnitus is also heard by other people and the most common cause are vascular anomalies.^2^ Subjective tinnitus is defined as the sound heard only by the patient. The pathology leading to tinnitus can be located anywhere from the ear canal to the hearing nervous center. Subjective tinnitus accompanied by sensorineural hearing loss are the most common symptoms of tinnitus secondary to acoustic trauma.^17^ Among the diseases that can cause subjective tinnitus are otosclerosis, tympanosclerosis, acoustic trauma, sudden hearing loss, stress, ototoxic drugs, Meniere's disease, head trauma, and acoustic neuroma.^18^ Some metabolic, neurological, psychological and pharmacological diseases can also be causes of subjective tinnitus.^19^

The etiopathogenesis of tinnitus has not yet been clarified. The development of tinnitus can often be due to a cochlear damage.^20^ In some studies, oxidative stress has been reported to disrupt the sensorineural epithelium of the labyrinthine, acoustic, and vestibular nerves.^20^, ^21^, ^22^ Some experimental studies reported that ROS can damage the cochlear sensory epithelium.^23^ Some others have reported an increase of oxidative stress in tinnitus patients.^24^ Oxidative stress plays an important role in the pathogenesis of tinnitus due to acoustic trauma. Excessive noise exposure, may cause ROS accumulation, which can lead to necrosis and apoptosis of the outer hair cells.^25^ Normally, the human cochlea contains antioxidant vitamins, glutathione and various enzymes against oxidative molecules. According to some reports, these antioxidants may not be detoxifying enough to play a role in protecting against acute trauma and some other ear diseases.^26^ Thus, sensorineural epithelium is at risk for ROS-induced lesions in cochlea. Oxidative stress has been reported to damage the labyrinth, acoustic and vestibular nervous system.^27^

Savastano et al., prescribed phospholipids and various vitamins as oral antioxidant therapy for a total of 31 patients with idiopathic unilateral tinnitus for 18 weeks. They were submitted to a pure tone audiometric evaluation, and the severity of ear tinnitus was determined by a visual analogue scale (VAS) 48 h before and after the treatment. There was a reduction of the tinnitus intensity with the antioxidant treatment.^28^ Similarly, Gopal et al., reported that the severity of tinnitus decreased after antioxidant therapy and concluded that antioxidant agents might be an option in tinnitus treatment.^29^ On the other hand, Polanski et al., reported that antioxidant agents had no benefits in the treatment of tinnitus.^30^

Neri et al., investigated the levels of malonaldehyde, 4-hydroxynonenal and myeloperoxidase enzymes, which are markers of oxidative damage, in their study of 44 patients with tinnitus and 25 healthy volunteers.^31^ In the patients with tinnitus, these 3 parameters were found to be higher than in the control group and the antioxidant enzyme glutathione peroxidase was found to be lower. According to the authors, this elevated oxidative stress may cause vascular endothelial dysfunction leading to a defect in the microcirculation of the inner ear. Based on these findings, oxidative stress was found to be important in tinnitus pathogenesis. Koc et al., studied 54 tinnitus patients and 60 health subjects. They reported that TIN and oxidative stress levels were statistically significantly higher in patients with tinnitus. TAS and PON levels were significantly lower in the tinnitus group than in the control group. They concluded that patients with tinnitus were exposed to oxidative stress.^24^ In our study, we found that TOS and OSI levels were statistically higher in tinnitus patients than in controls. We also found high levels of SPEA in patients with tinnitus.

Aubert et al., in an experimental study carried out on frogs, found that the injection of phenazine methasulfate into the perilymphatic region caused an increase in ROS formation and deterioration of endolymph secretion. They reported that the addition of trimethasine to the perilymphatic region was protective against harmful effects of ROS.^22^ These results indicate that the beneficial effect of trimetazine observed during tinnitus treatment may be due to antioxidant properties.

Prolidase plays an important role in the regulation of collagen biosynthesis and degradation. The prolidase enzyme is the only enzyme that catalyzes the intracellular rapid hydrolysis of proline and hydroxyproline that occurs at the end of collagen catabolism. So, the collagen metabolism can be determined by the SPEA levels.^32^ In a study conducted in rats, it was shown that exposure of proline to rats’ brains reduces the antioxidant potential and induces oxidative stress.^33^

Some studies in the literature have reported that there is a direct relationship between tinnitus and various psychological disorders.^34^ In our literature review, we found that the relationship between tinnitus and prolidase enzyme has not been previously investigated. However, it has been reported that the activity of prolidase enzyme increases in diseases such as anxiety disorder, depression, bipolar disorder, which are frequently associated with tinnitus. For this reason, we added the enzyme prolidase in our investigation.

Simsek et al., observed that the incidence of anxiety disorder and depression was significantly increased in patients with subjective tinnitus.^35^ Ercan et al. reported that oxidative stress index and prolidase enzyme activity were increased in anxious patients compared to the control group. They also reported that levels of oxidative stress severity may be related to SPEA enzyme levels, thus indicating that SPEA levels may be indicative of disease activity in anxiety disorders.^12^

One limitation of this study was the small size of both, patient and control groups. Regardless, we still believe that investigating the relationship between tinnitus symptoms scores and SPEA and OSİ levels may be useful.

## Conclusion

We found that total oxidant status, oxidative stress index and serum prolidase enzyme activity levels were statistically higher in patients with tinnitus than in controls. There was no significant difference between the tinnitus and control groups in terms of total antioxidant status levels. These findings suggest that tinnitus patients were found to be exposed to more oxidative stress. Elevated serum prolidase enzyme activity and oxidative stress index levels may have a role in the pathogenesis of tinnitus. There is a need for more extensive and comprehensive work in this area.

## Funding

This study was funded by Hitit University Scientific Research Projects.

## Conflicts of interest

The authors declare no conflicts of interest.
